# A Pilot Study of Facial Nerve Stimulation on Cerebral Artery Vasospasm in Subarachnoid Hemorrhage Patients

**DOI:** 10.1109/JTEHM.2019.2937121

**Published:** 2019-09-26

**Authors:** Daniel San-Juan, Marco A. Zenteno, Dania Trinidad, Franklin Meza, Mark K. Borsody, María De Monserrat Godínez García, María Cecilia Martínez, Fernando Castro Prado, Emilio Sacristan

**Affiliations:** 1 Department of Clinical NeurophysiologyNational Institute of Neurology and NeurosurgeryMexico City04257Mexico; 2 Department of Neuroendovascular TherapyNational Institute of Neurology and NeurosurgeryMexico City04257Mexico; 3 NeuroSpringDoverDEUSA; 4 National Center for Medical Imaging and Instrumentation ResearchUniversidad Autónoma Metropolitana-Iztapalapa 27788 Mexico City04257Mexico; 5 Specialties Hospital, XXI Century National Medical CenterIMSS, Angeles Pedregal HospitalMexico City04257Mexico

**Keywords:** Cerebral artery vasospasm, facial nerve, magnetic stimulation, cerebral blood flow

## Abstract

Background: The objective of this pilot study was to assess the safety and efficacy of VitalFlow stimulation in aneurysmal subarachnoid hemorrhage (aSAH) patients with vasospasm for the purpose of guiding the design of larger, controlled studies in vasospasm patients, a largely untreated condition [1]. Methods: Six patients with angiographic vasospasm developing post-aSAH were treated with VitalFlow stimulation. Digital subtraction angiograms were obtained at the time of diagnosis (baseline) and then 30 minutes post-stimulation. A single 2-minute period of stimulation was delivered to the patients using parameters previously shown to be safe, tolerable, and effective at increasing cerebral blood flow (CBF) in healthy volunteers. Results: VitalFlow stimulation improved tissue perfusion as assessed by parenchymography and reversed the constriction of vasospastic arteries. Two patients had only partial improvement and so were treated with intraarterial nimodipine after VitalFlow stimulation, whereas four patients had complete resolution of the vasospasm after VitalFlow stimulation per the treating neuroendovascular surgeon’s evaluation. Clinical examination showed improvement in Hunt and Hess Scale scores assessed post-stimulation. Conclusions: Non-invasive magnetic stimulation of the facial nerve with the VitalFlow stimulator appears to be a safe and effective means to reverse angiographic vasospasm in aSAH patients. Clinical Impact: This study provides Class IV evidence that non-invasive magnetic stimulation of the facial nerves reduce angiographic vasospasm in aSAH patients.

## Graphical abstract

This pilot study assessed VitalFlow stimulation in aneurysmal subarachnoid hemorrhage (aSAH) patients with vasospasm for the purpose of guiding the design of larger, controlled studies in patients with vasospasm, a largely untreated condition. Results in six patients showed that stimulation improved tissue perfusion as assessed by parenchymography and reversed the constriction of vasospastic arteries. This study provides Class IV evidence that non-invasive magnetic stimulation of the facial nerves reduces angiographic vasospasm in aSAH patients.

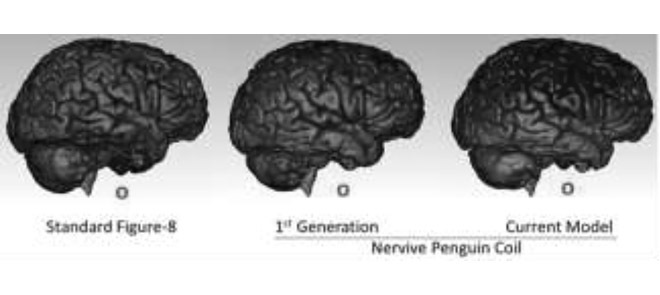

## I. Background

We report the results of a pilot study of a new treatment for conditions of brain ischemia such as vasospasm, a complication of aSAH in which one or more cerebral arteries constrict in response to blood products. No effective treatment option currently exists for vasospasm because it can affect both large and small arteries alone or in combination. We have created a non-invasive medical device called the VitalFlow stimulator that rapidly dilates the cerebral arteries and increases cerebral blood flow (CBF) by activating the facial nerves with pulsed magnetic fields. The autonomic components of the facial nerve (i.e., the petrosal branches) are well-known to dilate the cerebral arteries by means of a parasympathetic action [2]–[15]. Direct electrical stimulation of the autonomic components of the facial nerve (hereafter simply “facial nerve”) has been shown to reverse angiographic measures of vasospasm in dogs and monkeys [16], [17] and similarly to improve other conditions of brain ischemia such as ischemic stroke [18], [19].

We confirmed these findings with an animal prototype VitalFlow stimulator, which increased CBF in normal animals [21] and restored lost CBF in dogs and rabbits with embolic ischemic stroke [22], [23]. A clinical prototype VitalFlow stimulator was subsequently assessed in 35 healthy volunteers for safety, tolerability, and efficacy at increasing CBF [24]. VitalFlow stimulation for 3 minutes in healthy volunteers caused only transient side-effects during stimulation and it increased CBF by 32 ± 6% (mean ± SEM).

In the pilot study reported in this manuscript, we used the clinical prototype VitalFlow stimulator to treat 6 aSAH patients who developed angiographic vasospasm at the National Institute of Neurology and Neurosurgery (NINN) of Mexico.

## II. Methods

### A. Study Design

This was an open-label study of safety and efficacy testing a single treatment with the VitalFlow stimulator, an unlabled medical device in clinical development (Nervive Inc; Akron, OH). No control group is needed or appropriate because this is a pilot study of an acute treatment of a slowly-progressive condition based upon immediate pre- and post-stimulation assessments of objective measures (angiograms, parenchymograms) and standard clinical scales (Hunt and Hess Scale, modified Rankin Scale). Furthermore, this pilot study is the equivalent of an Early Feasibility Clinical Study that, under U.S. FDA regulations, would be limited to 10 patients [25]. A larger cohort of patients would thus not be appropriate for a pilot study.

### B. Standard Protocol Approvals, Registrations, and Patient Consents

The study was conducted under the ethical and regulatory approval of the NINN Bioethics and Research Committee, which is responsible for human experimentation at the NINN. All patients or their direct family members provided written informed consent as per the NINN institutional requirements. This study is listed on the public trials registry of clinicaltrials.gov as NRV_PI_01_18.

### C. Participants

Six subjects were recruited consecutively from the Department of Neuroendovascular Therapy at the NINN. Aneurysmal rupture was confirmed as the cause of the SAH and the aneurysm was treated with endovascular coiling or surgical clipping before VitalFlow stimulation was permitted. Other inclusion included adults aged ≥18 years old, any race, either gender, aneurysmal subarachnoid hemorrhage (non-traumatic), and angiographic vasospasm confirmed by catheter-based cerebral angiography. Exclusion criteria included: any other untreated cerebrovascular disease including pseudoaneurysm and arteriovenous malformation; hyperthermia at the time of stimulation; NIHSS score ≥ 20 including new focal neurological deficits attributable to vasospasm; angiographic vasospasm limited to posterior circulation; pregnancy; previous seizure without antiepileptic treatment; metallic foreign bodies or other implants in head or neck except MRI-compatible aneurysm coils and clips; unstable or uncontrollable medical conditions; previous history of glaucoma; or cranial nerve palsies or temporal bone fractures.

As expected for the patient population of the NINN (a quaternary referral center for the entirety of Mexico), all but one patient lived outside of Mexico City, which accounts for the irregularity in follow-up post-hospitalization.

### D. Pre- and Peri-Stimulation Procedures

aSAH patients were managed in the 22-bed neuro-ICU under a joint team of anesthesiologists and neuroendovascular surgeons. All patients received prophylaxis for vasospasm with oral nimodipine and the maintenance of euvolemia [1]. All patients were evaluated daily with transcranial Doppler ultrasonography (TCD) for escalating flow velocities, which we assessed according to the criteria of Sloan et al [26] as “possible” or “presumed definite” vasospasm. Patients were evaluated with digital subtraction angiography (DSA) to assess for angiographic vasospasm according to standard clinical practice at the NINN [27]. If the patient demonstrated angiographic vasospasm, he or she was stimulated with the VitalFlow device and then repeat DSA studies were obtained at 30 minutes post-stimulation. Intraarterial nimodipine therapy was allowed at the neuroendovascular surgeon’s discretion if he or she considered the response to VitalFlow stimulation to be partial or incomplete, but no additional treatment was allowed until after the repeat DSA study 30 minutes post-stimulation.

During VitalFlow stimulation, the patients were maintained on propofol and fentanyl sedation typically used for the angiography procedure and were monitored by anesthesiologists with vital signs being recorded each minute. Hunt and Hess Scale scores were obtained before sedation and after sedation.

### E. Digital Subtraction Angiography (DSA)

DSA was performed on an Artis Zee / Zeego robotic arm angiography system and VB21C Syngo Workplace©post-processing station (Siemens; Erlangen, Germany). Standard DSA images were used to qualitatively identify areas of likely angiographic vasospasm that were quantified using Osirix Lite (Pixmeo). An artery segment was considered vasospastic only if its diameter was reduced by 50% in comparison with the immediately upstream segment of normal artery. Artery diameter measures were placed by agreement of two investigators (DSJ, DT) and under review by a third investigator (MAZ), who were blinded to the order of the neuroimages.

### F. VitalFlow Stimulation

Facial nerve stimulation was performed with a modified transcranial magnetic stimulator (Neuro-MS/D Therapeutic; Neurosoft, Ivanovo, Russia) equipped with two custom-designed fluid-cooled magnetic stimulation coils. Stimulation power was set at 80% of the stimulus generator’s maximum output, which corresponds to approximately 1.6 T magnetic field strength at the coil surface. Stimulation was administered with $280~\mu $sec biphasic pulses delivered at 10 Hz for a 2-minute period. No ramp-up was employed. Stimulation coils were placed bilaterally on the head against the ears and were fixed into position with lockable arms based on the headrest. The final positioning of the stimulation coils was guided by MRI neuronavigation that aimed the central focus of the magnetic field at the geniculate ganglion region of the facial nerve, as described previously [22].

**FIGURE 1.**
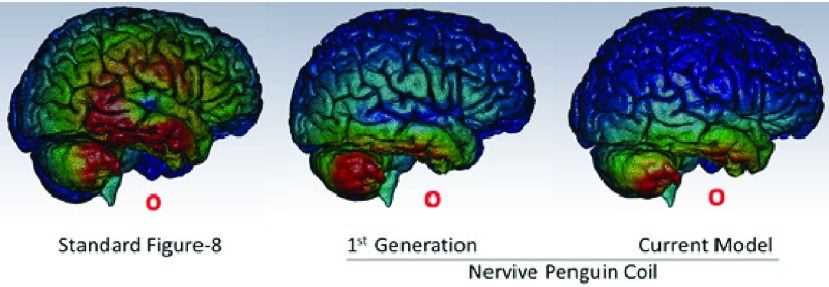
Induced electrical current in tissue models of the human brain comparing the MagStim 6.5 cm figure-8 coil against Nervive’s proprietary penguin coil design. Red shift = increasing current density.

VitalFlow stimulation coils are of a novel, proprietary design specifically intended for use in man based according to the human head anatomy. Based on computer modeling of induced electrical currents in tissue models of 3 human heads, the VitalFlow stimulation coil design redistributes the generated magnetic field in a manner that reduces brain exposure by more than 80% in comparison with commercially-available figure-8 transcranial magnetic stimulation coils (FIGURE 1).

### G. Post-Stimulation Procedures

The patients were monitored in the neuro-ICU and received the standard care of the treatment of aSAH [1]. Patients routinely were kept in-hospital at a lower level of acuity after their ICU hospitalization. Follow-up post-hospitalization was conducted by one of the study investigators (MAZ) at times that were determined in by the patient’s ability to travel back to the NINN from remote locations in the countryside, if possible. Modified Rankin scores were obtained at all follow-up visits.

### H. Analysis

DSA were assessed qualitatively and quantitatively for improvement in the diameter of vasospastic large cerebral arteries in comparison with the baseline angiogram. Quantitative measures of large artery vasospastic segments were performed by one of the authors (MKB) who was not involved in the experiments or in patient care and was subsequently confirmed by all the authors. Percentage constriction was calculated at the point of maximum narrowing divided by the immediate upstream segment of the artery that was not vasospastic nor exhibiting branches. Parenchymograms were evaluated qualitatively. Statistical analyses were not appropriate for this dataset. Deidentified data is available for review through the corresponding author.

## III. Results

We included six patients, 3 males and 3 females, who on average were 43.5 (19–53) years-old. A Supplemental Document to this manuscript summarizes the angiographic and clinical findings measurements.

Case #1: 19-year-old female with no past medical history. The patient presented to the NINN with a severe headache and a generalized seizure, followed by somnolence and confusion. Her exam also demonstrated a right Babinski reflex and neck rigidity but no motor or sensitive deficit. A head CT scan demonstrated Fisher grade III aSAH. DSA on Day 1 (the day of hemorrhage) showed a 4 mm aneurysm on the left posterior communicating artery that was secured by coil embolization. On Day 3, TCD showed possible vasospasm of the left MCA (mean velocity 155 cm/sec). By Day 4, TCD showed possible vasospasm in both MCAs (left mean velocity 161 cm/sec; right mean velocity 147 cm/sec).

**FIGURE 2.**
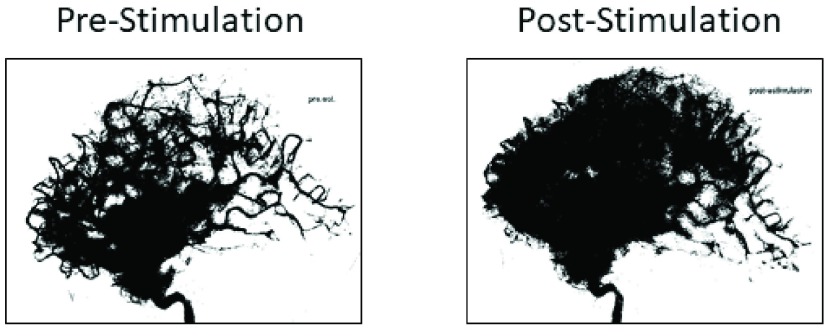
Case #1. Parenchymograms before and after VitalFlow stimulation, lateral view. Immediate pre-stimulation (left) versus 30-min post-stimulation (right).

On Day 5, the patient again underwent DSA that showed vasospasm of the left internal carotid artery. VitalFlow stimulation was administered with improvement of the parenchymogram (FIGURE 2); no further treatment was given. On Day 9, sedation and mechanical ventilation were removed and the patient was discharged on Day 17 without neurological deficits. The patient has been evaluated at 2, 6, and 9 months’ post-hospital discharge and was without neurological impairment on examination.

Case #2: 45-year-old female with a history of hypertension and 20 pack-year cigarette smoking.

The patient presented to an outlying hospital after a loss of consciousness and incontinence. She was found to have SAH and hydrocephalus; her history suggested she may have had a sentinel hemorrhage 2 months earlier. She could not be transferred to the NINN until Day 12 due to the limited ambulance services. Upon her arrival at the NINN, the patient’s neurological examination showed somnolence and confusion, but no focal motor or sensory deficit other than hyperreflexia in the lower limbs. Head CT scan confirmed Fisher grade IV SAH, and CT angiography demonstrated a 6 mm left posterior communicating artery aneurysm.

**FIGURE 3.**
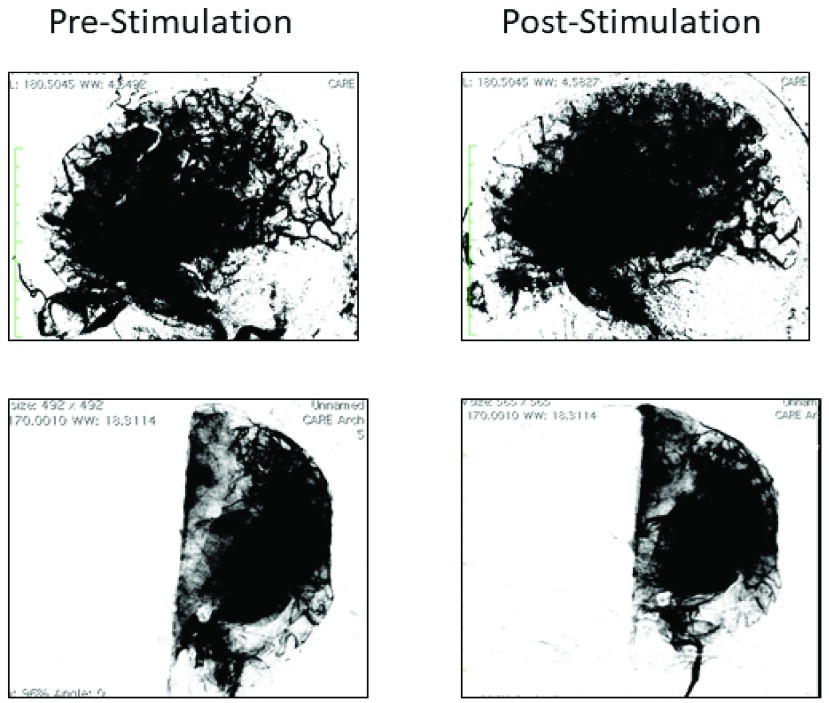
Case #2. Parenchymograms before and after VitalFlow stimulation, lateral (top panels) and anterior-posterior (bottom panels) views. Immediate pre-stimulation (left) versus 30-min post-stimulation (right).

On Day 13, the patient underwent DSA and the aneurysm was treated with coil embolization. She was then transferred to the neuro-ICU on mechanical ventilation and sedation. She underwent ventriculostomy on Day 14 and was treated with induced hypertension. On Day 14, she had another DSA study demonstrating severe vasospasm in the left middle cerebral artery territory and at that time she was treated with VitalFlow stimulation. The vasospasm partially resolved after stimulation per the neuroendovascular surgeon’s evaluation (FIGURE 3), and intra-arterial nimodipine was then administered with additional improvement. By Day 15, steadily increasing TCD velocities led us to repeat DSA, which demonstrated moderate vasospasm on the left middle cerebral artery that was treated with intra-arterial nimodipine to resolution, as confirmed by CT angiography on Day 19. Sedation and ventilation were discontinued on Day 20 and the patient was without focal neurological deficit. The ventriculostomy was removed on Day 22 and the patient was discharged on Day 23. Follow-up at 3 months noted only an apathetic personality in the patient, which was not observed upon examination at 6 months.

Case #3: 50-year-old male with no medical history.

The patient presented with a severe headache associated with nausea and vomiting. Head CT obtained at an outlying hospital demonstrated Fisher grade III SAH, and the patient was transferred to the NINN. Upon admission, he was normotensive and had reduced visual acuity bilaterally without any other focal neurological deficits. DSA performed on Day 11 demonstrated a 3 mm aneurysm on the left internal carotid artery. The aneurysm was clipped surgically on Day 12 without any complications.

**FIGURE 4.**
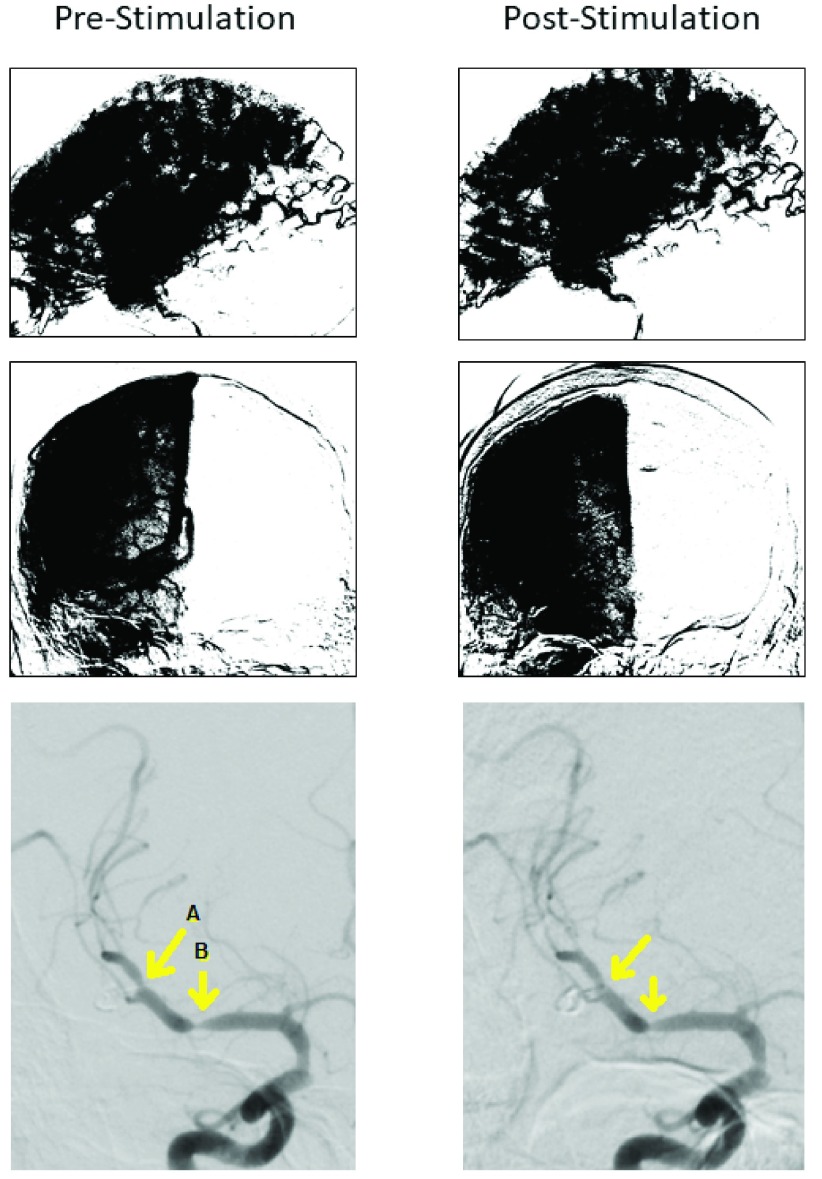
Case #3. Parenchymograms before and after VitalFlow stimulation, lateral (top panels) and anterior-posterior (middle panels) views. Anterior-posterior view DSA demonstrating large artery vasospasm of the right middle cerebral artery (bottom panels). Arrow A: 45% diameter pre-stimulation, 63% diameter post-stimulation. Arrow B: 38% diameter pre-stimulation, 50% diameter post-stimulation. Immediate pre-stimulation (left) versus 30-min post-stimulation (right).

On Day 13, DSA showed obliteration of the aneurysm and vasospasm on left middle cerebral artery. The patient was then treated with VitalFlow stimulation with partial effectiveness per the neuroendovascular surgeon’s evaluation (FIGURE 4), including vasospastic segments of the right MCA (distal vasospastic segment: 45% diameter pre-stimulation, 63% diameter post-stimulation; proximal vasospastic segment: 38% diameter pre-stimulation, 50% diameter post-stimulation). Intraarterial nimodipine was then administered, leading to complete resolution of the large artery vasospasm, and no vasospasm was identified on CT angiograms completed on Day 15. The patient was discharged from the hospital at Day 20 without neurological impairment.

Case #4: 44-year-old male with a history of alcoholism, 25 pack-year cigarette smoking, and methamphetamine use.

The patient presented with lightheadedness causing him to fall with a brief period of unconsciousness, after which he was taken for evaluation to an outlying hospital where he was treated symptomatically for a severe headache and discharged. He was reevaluated at the NINN on Day 7 because of persistent headache and found to have right mydriasis without other neurological findings. A head CT scan performed at that time showed edema and hemorrhagic contusion over the right temporal lobe and right hemispheric edema associated with Fisher grade III SAH; CT angiography demonstrated only a right middle cerebral artery aneurysm of 2 mm diameter.

**FIGURE 5.**
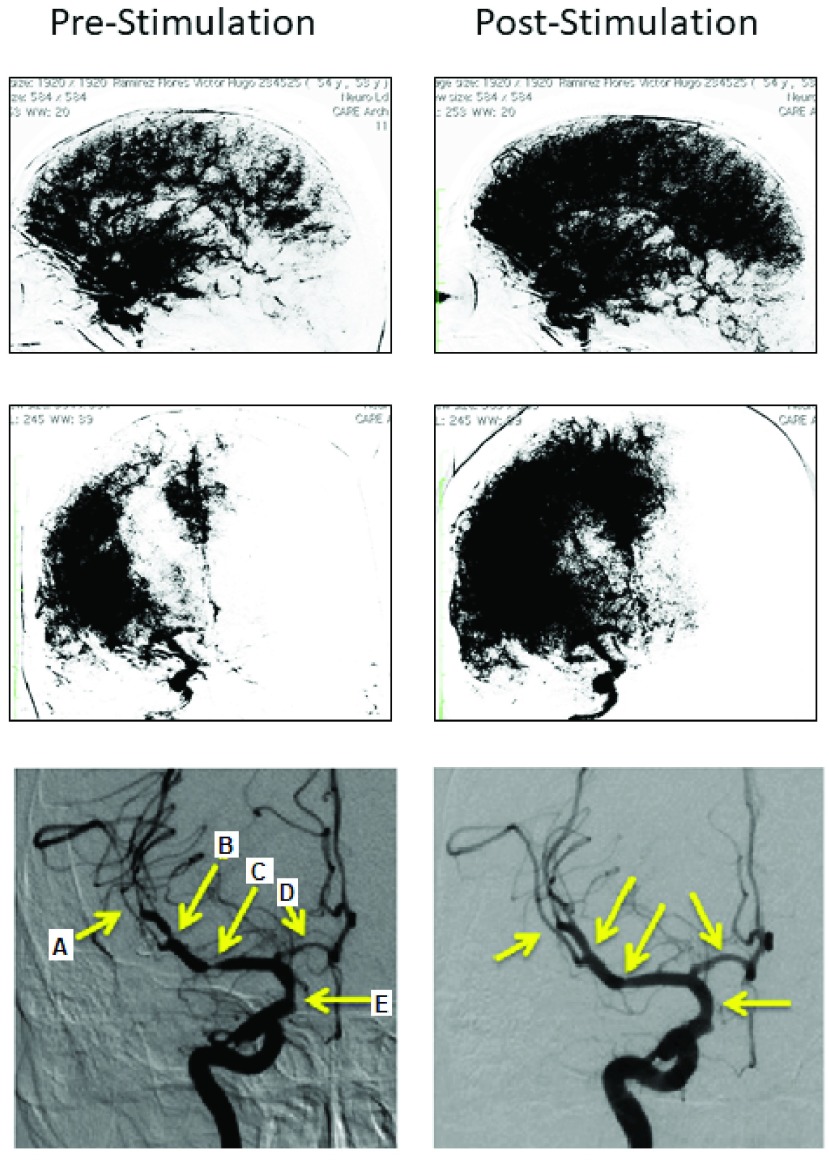
Case #4. Parenchymograms before and after VitalFlow stimulation, lateral (top panels) and anterior-posterior (middle panels) views. Anterior-posterior view DSA demonstrating large artery vasospasm in multiple cerebral arteries (bottom panels). Arrow A: 38% diameter pre-stimulation, 50% diameter post-stimulation. Arrow B: 42% diameter pre-stimulation, 67% post-stimulation. Arrow C: 29% diameter pre-stimulation, 75% diameter post-stimulation. Arrow D: 30% diameter pre-stimulation, 63% diameter post-stimulation. Arrow E: 47% diameter pre-stimulation, 67% diameter post-stimulation. Immediate pre-stimulation (left) versus 30-min post-stimulation (right).

DSA on Day 8 confirmed the aneurysm and on Day 9 the aneurysm was surgically clipped without complication and with resolution of the mydriasis. However, repeat DSA on Day 10 to confirm aneurysm obliteration showed severe vasospasm in the right anterior cerebral and right middle cerebral arteries. Multifocal vasospasm in large cerebral arteries was also evident. Immediately thereafter, the patient underwent VitalFlow stimulation leading to partial resolution of the vasospasm per the neuroendovascular surgeon’s evaluation (FIGURE 5), which was then completely resolved with intraarterial nimodipine treatment. The patient was discharged to home on Day 18. The last follow-up on Day 24 found the patient to be without any neurological impairment.

Case #5: 50-year-old female with no medical history. The patient presented with headache and neck pain that increased in severity over several hours, leading to loss of vision on her right eye and several episodes of vomiting by Day 2. On Day 4, she lost consciousness for a period of 5 minutes, and afterward her family had her evaluated by a general physician who provided her analgesic medications for the headache. However, on Day 9, the patient suffered a generalized seizure. It was not until Day 16 that the patient sought medical attention at an outlying hospital where CT and CT angiogram scans demonstrates Fisher grade IV SAH and a 15 mm aneurysm on the left anterior cerebral artery.

**FIGURE 6.**
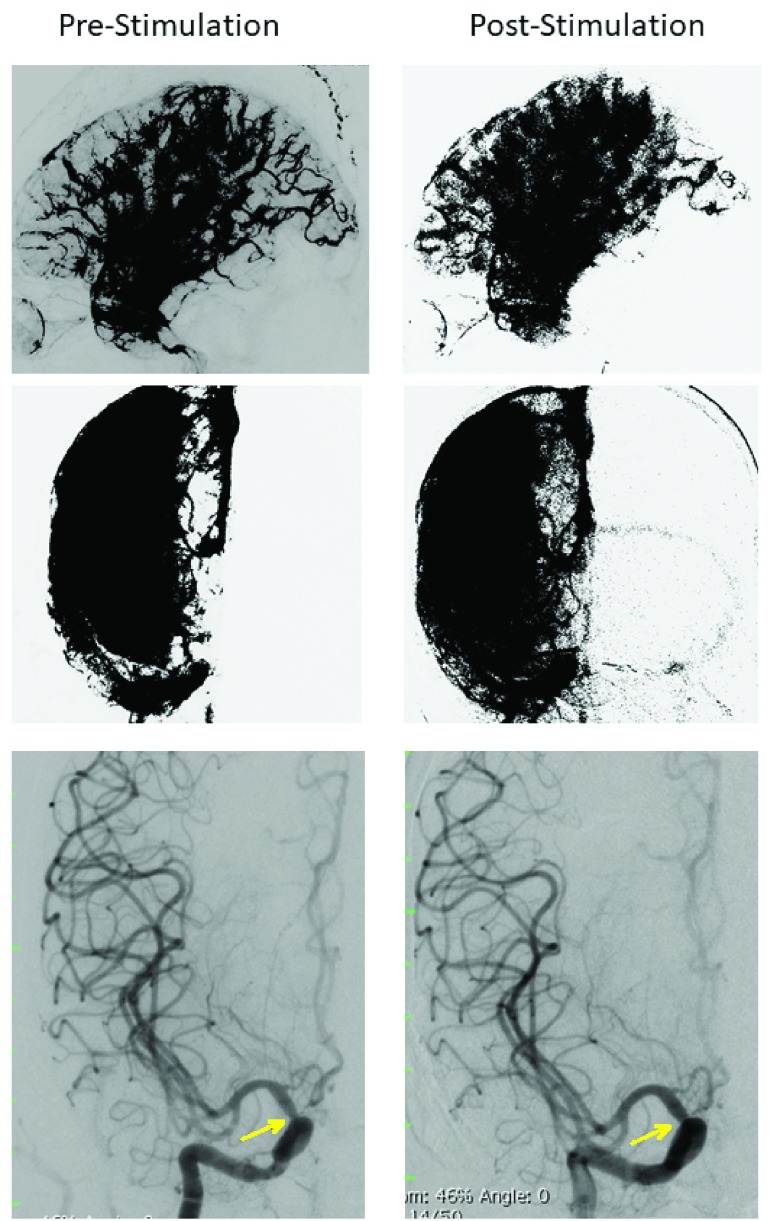
Case #5. Parenchymograms before and after VitalFlow stimulation, lateral (top panels) and anterior-posterior (middle panels) views. Anterior-posterior view DSA demonstrating large artery vasospasm in the internal carotid artery (bottom panels). At arrow: 63% diameter pre-stimulation, 85% diameter post-stimulation. Immediate pre-stimulation (left) versus 30-min post-stimulation (right).

On Day 17, the patient was transferred to the NINN. Upon admission, her physical examination showed a stuporous state and neck rigidity. The patient received a ventriculostomy on Day 18 without any complications, followed the same day by DSA demonstrating the aneurysm and severe segmental vasospasm of the internal carotid artery. Because of the patient’s unstable condition, the aneurysm could not be clipped until Day 27, at which time it was completed without complication. On Day 30, repeat DSA (FIGURE 6) showed worsening vasospasm that was treated with VitalFlow stimulation, improving the parenchymogram and vasospastic segment of the internal carotid artery (63% diameter pre-stimulation, 85% diameter post-stimulation). No further treatment for vasospasm was given. The patient’s remaining hospitalization was unremarkable and she was discharged to a nursing home on Day 45 with a gastrostomy and tracheostomy.

Case #6: 53-year-old male with hypertension and 15 pack-year smoking history.

The patient presented with rapidly worsening headache and blurred vision followed by a brief loss of consciousness. He was taken to an outlying hospital where he was diagnosed with uncontrolled hypertension and was discharged on an antihypertensive medication. Due to the persistence of the headache, the patient consulted a private neurologist who obtained a head CT and CT angiogram on Day 8. CT head scan showed a Fisher grade III SAH and 3 mm anterior communicating artery aneurysm. The patient was then referred to the NINN. At admission on Day 10, he was somnolent and disoriented, and his physical examination showed neck rigidity and a left Babinski reflex. On this same day, he underwent endovascular coil embolization of the aneurysm.

**FIGURE 7.**
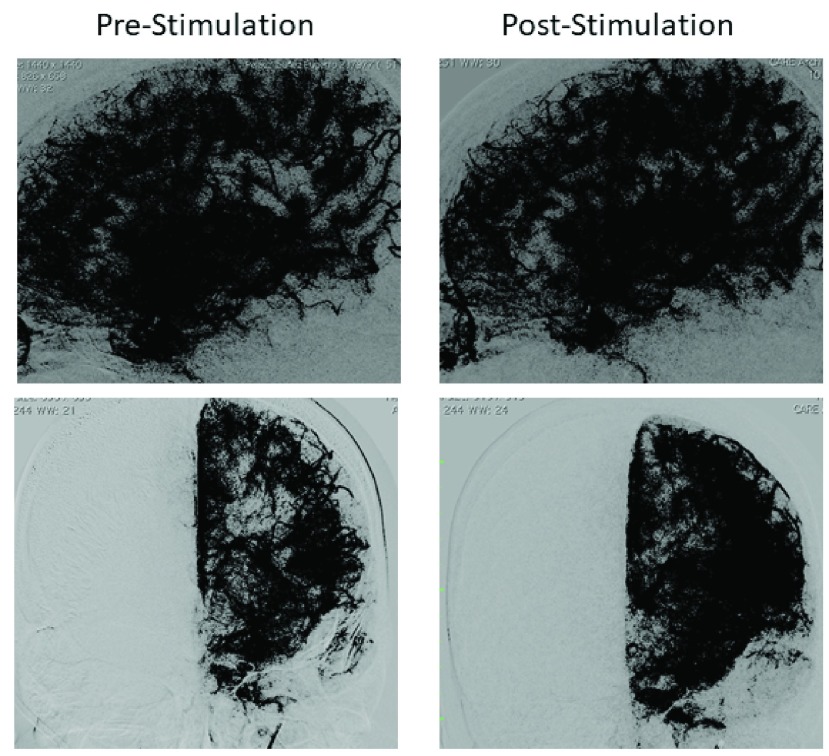
Case #6. Parenchymograms before and after VitalFlow stimulation, lateral and anterior-posterior views. Immediate pre-stimulation (left) versus 30-min post-stimulation (right).

On Day 11, the patient underwent follow-up DSA that demonstrated small vessel vasospasm in the left anterior cerebral artery distribution. The patient was then treated with VitalFlow stimulation leading to complete resolution of the vasospasm (FIGURE 7). Subsequent TCD monitoring over 4 days was normal. The patient was discharged on Day 16 without neurological impairment.

## IV. Discussion

As a novel treatment for brain ischemia, the VitalFlow stimulator is based on two well-established principles: first, that the autonomic components of the facial nerve dilate the cerebral arteries and increase CBF; and, second, that pulsed magnetic energy can activate neural structures such as the facial nerve. The combination of these principles allows for a medical device that can be non-invasively applied to a patient with brain ischemia as a rapidly-administered treatment. Herein we tested the VitalFlow stimulator in patients with delayed cerebral artery vasospasm, a complication of aSAH that causes brain ischemia. In a group of 6 patients with angiogram-confirmed vasospasm, the VitalFlow was able to reverse the vasospasm in part or entirely, restoring CBF to the ischemic brain as is evidenced by qualitative improvements in the parenchymograms and increased calibers of vasospastic large arteries. VitalFlow stimulation, together with intraarterial nimodipine treatment in 2 patients, also improved Hunt and Hess Scale scores in every patient. We believe the change in CBF is likely directly impacting the patients’ clinical condition given the nature of vasospasm causing brain ischemia. Additionally, VitalFlow stimulation appeared to be safe in the vasospasm patients, much as has been shown in a study of 35 healthy volunteers who were stimulated with parameters comparable to those used in this pilot study [24]. No adverse events or tolerability issues were reported by the vasospasm patients, who notably were sedated at the time of VitalFlow stimulation.

Based on our preclinical research, we did not expect the vasodilation and increase in CBF caused by VitalFlow stimulation to last more than a few hours [21], [24], but the current results suggest it may be effective for longer periods. On the other hand, given the general safety and tolerability we observed in this pilot study, we see no reason that repeated VitalFlow stimulation cannot be administered in future studies. Indeed, doing so may have an additive effective, as suggested by experiments in normal pig subject to repeated VitalFlow stimulation [24].

Moreover, the partial effect of VitalFlow stimulation that we observed in cases #2 and #3 may reflect the slow, progressive vasodilation that occurs after magnetic facial nerve stimulation. This gradual build-up of vasodilation was observed in our earliest pilot experiments with sheep and dogs 21 and it was subsequently confirmed in pigs 24 but to date it has not been evaluated in man. It may be that the 30 minute follow-up period reflects the early part of the vasodilatory effect of VitalFlow stimulation, and that further resolution of the vasospasm would have been observable had we waited longer to perform the post-stimulation DSA. Future clinical study designs will also need to consider this factor with repeated measures of CBF.

A small sample size and the open-label design are inherent limitations to a pilot study such as this one, which has as its purpose to enlighten the design of a larger, controlled clinical study. Nevertheless, we intend to pursue additional clinical testing involving a larger cohort and a control group, and to that end we believe the results presented herein support the inclusion of repeated VitalFlow stimulation and later CBF measurements past the 30-minute post-stimulation time point.

## V. Conclusions

Non-invasive magnetic stimulation of the facial nerve with the VitalFlow stimulator appears to be a safe and effective means to reverse angiographic vasospasm in aSAH patients.
